# Cleomaceae: diversity and potential of a model family for studies on the evolution of photosynthesis

**DOI:** 10.1111/plb.70130

**Published:** 2025-11-26

**Authors:** P. Falquetto‐Gomes, D. F. Parma, J. Souza‐Isabel, W. E. B. Barrios, A. P. M. Weber, W. L. Araújo, A. Nunes‐Nesi

**Affiliations:** ^1^ National Institute of Science and Technology on Plant Physiology under Stress Conditions, Departamento de Biologia Vegetal Universidade Federal de Viçosa Viçosa Minas Gerais Brazil; ^2^ Institute of Plant Biochemistry, Cluster of Excellence on Plant Science (CEPLAS) Heinrich Heine University Düsseldorf Germany

**Keywords:** Brassicales, Cleomaceae, hercogamy, photosynthesis, pollination ecology

## Abstract

Understanding the physiological and molecular mechanisms by which plants adapt to environmental factors is essential for improving crop production and protecting biodiversity amid rapid anthropogenic climate change. The Cleomaceae is a family that stands out for its potential to study areas including floral diversity, species richness, and C_4_ photosynthesis. Its close relationship to the Brassicaceae allows for comparisons with *Arabidopsis thaliana*, which will lead to new knowledge that can be transferred to other species, including crops. This proximity paves the way for the investigation of monosymmetric and polysymmetric differences in flowers of the Brassicaceae. The rich variety of Cleomaceae floral forms represents a little studied but highly promising resource for understanding the evolution of key features that influence pollination. Additionally, Cleomaceae contain high concentrations of flavonoids, terpenoids, tannins, alkaloids, saponins, and anthocyanins, which could contribute to pharmaceutical discoveries and new health treatments. They also have significant potential in elucidating tolerance mechanisms to biotic and abiotic stresses, and can be consumed as food, although not traditionally cultivated. This review describes and discusses opportunities to advance research in various areas using Cleomaceae. Despite promising prospects, effective functional techniques to elucidate the diversity within this group are lacking.

## Graphical abstract

The Cleomaceae family integrates floral diversity, physiological plasticity, and the evolution of C_4_ photosynthesis, serving as an important model to investigate plant adaptation and evolutionary innovation.

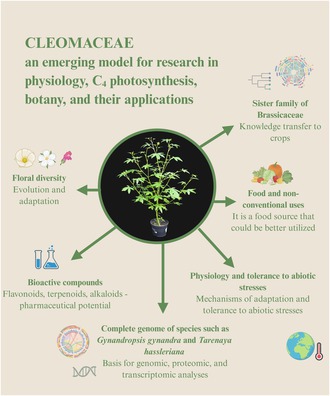

## INTRODUCTION

Cleomaceae is a small family of 27 formally described genera, comprising about 270 species (Stevens & Davis 2005; Bayat *et al*. 2018; Saunders *et al*. 2024). This family has a worldwide cosmopolitan distribution (Stevens & Davis 2005), originating in central Asia (Feodorova *et al*. 2010), and with a center of diversity in the Americas. Cleomaceae species have diverse habits (from herbaceous to shrub), monosymmetric flowers with a ground plan of four sepals, four petals, generally six stamens, and a bicarpellate gynoecium (Stevens & Davis 2005). The flowers are diverse in shape, size, and colour, as well as in the presence and size of the nectariferous disc, characteristics that influence the type of pollination (Stevens & Davis 2005).

The family is phylogenetically close to Brassicaceae, which includes the model species *Arabidopsis thaliana* (Hall *et al*. 2002; Stevens & Davis 2005; Hoang *et al*. 2023). Consequently, the Cleomaceae, distinguished by extensive physiological, anatomical, and morphological diversity, has become pivotal in ecological and evolutionary research. These studies have examined floral morphology and development (Nozzolillo *et al*. 2010; Patchell *et al*. 2014; Saunders *et al*. 2024), evolution of C_4_ photosynthesis (Feodorova *et al*. 2010; Van Den Bergh *et al*. 2014; Parma *et al*. 2022), pollination biology (Parma *et al*. 2021; Parma *et al*. 2023), and comparative genomics (Newell *et al*. 2010; Hoang *et al*. 2023).

Genomic studies of *Gynandropsis gynandra* identified significant whole‐genome duplications that are shared with C_3_ relatives, such as *Tarenaya hassleriana*. *G. gynandra* retains more copies of genes related to C_4_ photosynthesis, with many exhibiting signs of positive selection. This suggests that gene duplication and retention have contributed to evolution of the C_4_ pathway (Hoang *et al*. 2023). However, research also indicates that the primary distinction lies in gene regulation. Despite the presence of comparable numbers of C_4_‐related genes in these two species, attributable to ancient duplications, *G. gynandra* exhibits higher precision and tissue‐specificity in expression of these genes. This refined regulation is fundamental for the transition from C_3_ to C_4_ photosynthesis, suggesting that evolutionary changes in gene expression control, together with gene duplication, drove the origin of the C_4_ trait in Cleomaceae (Van Den Bergh *et al*. 2014). Consequently, both genomic events and regulatory modifications have complementary roles in this key physiological adaptation.

The current understanding of Cleomaceae remains disproportionately reliant on a limited number of species, e.g., *Gynandropsis gynandra*, *Tarenaya hassleriana*, and *T. spinosa*. The lack of studies on other members of Cleomaceae may be related to the scarcity of fundamental research, such as (i) floristic surveys and taxonomy – collection, ecological distribution, and identification; (ii) pollination studies; (iii) seed germination, considering that some species have dormancy and low viability; (iv) karyotyping; and (v) genome information. In light of this, studies in these areas have been increasing, as well as their scope for New World species, which had been little explored.

In summary, the Cleomaceae family has enormous potential to increase our knowledge in several areas of plant science (Van Den Bergh *et al*. 2014; Bayat *et al*. 2018; Parma *et al*. 2021; Parma *et al*. 2022; Hoang *et al*. 2023; Saunders *et al*. 2024). In this review, we have compiled studies on the Cleomaceae family across diverse fields and integrated this information to identify key evolutionary traits. In doing so, this work highlights the value of synthesizing multidisciplinary data and underscores the importance of cross‐disciplinary approaches in advancing understanding of Cleomaceae. By consolidating current knowledge, this review intends to facilitate future investigations and promote deeper exploration of the Cleomaceae family.

## HISTORY AND CLASSIFICATION

Taxonomic studies on the Cleomaceae family are relatively common. In this sense, the description of some genera that were included in the Capparaceae subfamily, Cleomoideae: *Cleomella*, *Peritoma*, *Polanisia*, *Dactylaena*, *Physostemon* (Eicheler, 1865; Pax & Hoffm 1936), *Podandrogyne* (Ducke, 1930), *Haptocarpum*, *Cristatella*, *Gynandropsis*, *Isomeris*, *Justago*, *Tetratelia* (Pax & Hoffm 1936; Kers 2003), *Oxystilis* and *Wislizenia* (Iltis, 1957; Keller 1979), are now placed in the Cleomaceae family. Eichler (1865) recognized only four genera within the Cleomae tribe (*Cleome* s.l., *Gynandropsis*, *Dactylaena* and *Physostemon*) and the genus *Cleome* s.l. was separated into three series based on habit. Subsequently, based on characteristics such as type of pubescence of the plant, presence or absence of spiniform stipules, large leaf bracts, as well as shape of flower buds and fruit, Heilborn (1931), seven groups were recognized within the series *Cleome* s.l. created by Eichler (1865). These studies provided the basis for the study of Pax & Hoffm (1936), which became the most comprehensive worldwide systematic treatment of *Cleome* and its close relatives.

Accordingly, *Cleome* s.l. (Cleomoideae) was divided into two supergroups (Old Word and New Word), two sections, four subsections, and six series, based on morphological characters, such as leaf division, habit, and stamen number (Pax & Hoffm 1936). However, this classification was largely based on characters that were taxonomically unreliable because of their large variability within the genus. Iltis (1952), in turn, considering characteristics of the seed, nectariferous disk, raceme morphology, floral aestivation and leaves, divided New World *Cleome* into two subgenera, 10 sections, and 17 series (Sánchez‐Acebo 2005; Inda *et al*. 2008).

Phylogenetic studies with the Cleomaceae family attempted to understand the circumscription of Capparaceae and Brassicaceae, since Cleomoideae (a subfamily of Capparaceae) positioned itself closer to Brassicaceae than Capparaceae (Hall *et al*. 2002). Thus, these studies found that Capparoideae, Brassicoideae, and Cleomoideae form distinct and well‐supported monophyletic clades. In that sense, some authors (Hall *et al*. 2002) recommended recognizing these subfamilies as three separate families, Capparaceae, Brassicaceae, and Cleomaceae. However, it was only in 2011 that Iltis *et al*. (2011) upgraded Cleomoideae to the family level, based on previous works on molecular phylogeny and morphological characterization. In this context, the Cleomaceae differ from the Capparaceae and Brassicaceae in capsular fruit without a septum, palm‐shaped compound leaves, and seeds decorated with a sharply invaginated forehead (Hall *et al*. 2002; Iltis *et al*. 2011).

After the recognition of Cleomaceae as a family, the focus became understanding the real circumscription of the genus *Cleome*, since it was very specious (about 200 species) and found to be polyphyletic in molecular phylogeny studies (e.g. Sánchez‐Acebo 2005; Hall 2008; Patchell *et al*. 2014). The most extensive research for this family, based on molecular phylogeny, aimed to understand the genus *Cleome* s.s., sampling the type species that, until then, had not been analysed in a phylogenetic context using five molecular markers (ndhF, ITS, rps3, matk, and ycF) and three genomes (nuclear, chloroplast, mitochondrial) (Patchell *et al*. 2014). After this study, several others emerged to delimit genera of this family and make them monophyletic (Barrett *et al*. 2017; Soares Neto *et al.* 2018; Roalson *et al*. 2015; Thulin & Roalson 2017). Thus, the family currently has 27 genera and about 270 species (Stevens & Davis 2005; Bayat *et al*. 2018; Saunders *et al*. 2024). However, it noteworthy that the number of genera and species is expected to grow as new research emerges in this field (Soares Neto *et al*. 2020).

## MORPHOLOGICAL EVOLUTION WITHIN CLEOMACEAE

In Cleomaceae, few studies have attempted to associate morphology with molecular phylogeny (Sánchez‐Acebo 2005). This association of traits is essential for classifying groups of Cleome to trace evolutionary aspects and trends. All morphological characteristics are important, but reproductive traits, such as pollen and seed morphology, are particularly informative for understanding the evolutionary history of Cleomaceae because they are related to the processes of reproduction and seed dispersal processes. Other reproductive traits that are usually evaluated include floral symmetry, the number of stamens, and petal shape (Sánchez‐Acebo 2005; Parma *et al*. 2021).

It has been demonstrated that Cleomaceae pollen is tricolporate and highly variable in ornamentation, ranging from echinate to reticulate, rugulate or verrucate‐psilate (Sánchez‐Acebo 2005). Thus, it is common to group species in the Cleomaceae based on the pollen features. Accordingly, the most basal groups of the family have reticulate or rugulate pollen and the most echinate‐derived pollen groups (Sánchez‐Acebo 2005). Consequently, studies of pollen morphology classified members of the Old World as having reticulate or rugulate pollen (e.g. North American group; Al‐Shehbaz 1985), African cluster, *Polanisia* sp., *G. gynandra* (Sánchez‐Acebo 2005), *C. viscosa*, *C. karachiriensis* (Riaz *et al*. 2019). Echinate ornamentation type of pollen is present in *Tarenaya*, Andean group, *Podandrogyne* sp., *Cleome stylosa*, *C. lechleri*, *C. anomala* and *C. moritziana* (Sánchez‐Acebo 2005), which suggests that echinate pollen may be derived from reticulate or rugulate pollen (Sánchez‐Acebo 2005). However, exclusively, *Melidiscus giganteus* has verrucate pollen (Sánchez‐Acebo 2005), and *C. brachycarpa* has spinulose ornamentation (Riaz *et al*. 2019).

Regarding seed morphology, Cleomaceae has several types of ornamentation, ranging from smooth (e.g., *Cleoserrata paludosa*; Sánchez‐Acebo 2005) to verrucose seeds. The *Tarenaya* species, such as *T. aculeata*, *T. crenopetala*, *T. dendroides*, *T. diffusa*, *T. eosina*, *T. hassleriana*, *T. horrida*, *T. microcarpa*, *T. parviflora*, *T. regnelli*, *T. rosea*, *T. spinosa*, *T. titubans*, *T. trachycarpa* and *T. virens* have reticulate ornamentation of the seeds, reticulate‐areolated with transversal, ribbed and alveolar ridges, in addition to the presence of an aryl in seeds of some species (Costa‐e‐Silva 2000; Parma *et al*. 2021). The seeds of *Melidiscus giganteus* are smooth (Sánchez‐Acebo 2005). Species of the genus *Podandrogyne* are characterized as having fleshy arils attached to the radicular claw, rounded seed shapes, relatively smooth surfaces, and generally larger seed size compared to other species in the family. In contrast (Saunders *et al*. 2024), *Rorida* species are among the smallest in Cleomaceae, with reddish seeds, and surfaces ranging from smooth to papillate. *Physostemon* seeds typically have prominently spiked surfaces (Saunders *et al*. 2024).

In Old‐World Cleomaceae, the seed typically splits open along a cleft, although the extent of dehiscence can be minimal in some species. In contrast, species in the New World group, such as *Tarenaya* spp. and *M. giganteus*, have a seed cleft that is predominantly closed by a membrane, which varies in thickness. Thus, while pollen and seed ornamentation patterns do not offer a clear synapomorphy for the entire Cleomaceae family due to numerous exceptions, these traits generally support the basal position of Old World species relative to New World species (Sánchez‐Acebo 2005), which is congruent with molecular phylogeny (Bayat *et al*. 2018). Therefore, further research is needed to better understand the potential of seed morphology in defining generic and species boundaries within Cleomaceae. Expanding the sampling at both species and genus level may help identify more consistent patterns (Saunders *et al*. 2024). Additionally, a more detailed classification of traits — such as the presence or absence of hairs, arils, and spines — could provide valuable data for future morphological analyses (Saunders *et al*. 2024). However, the current limitation in the number of seeds analysed per species and genus poses a challenge to drawing more precise conclusions. The morphological variation observed among groups may be underestimated, and only through broader sampling will it be possible to fully grasp the true diversity and potential evolutionary relationships within the Cleomaceae family (Parma *et al*. 2021).

Besides pollen and seed traits, the morphology of floral nectaries also adds to the diversity within the Cleomaceae and offers insights into its evolution and taxonomic description. The floral nectaries in Cleomaceae display significant variations in terms of location, size, shape, and secretory mechanism. They typically consist of receptacles located between the perianth and stamens, containing the nectary parenchyma with nectarostomata (Zenchyzen *et al*. 2024). Recently, a study on the morphology of nectaries in *C. violacea* revealed a notable degree of conservation between the Cleomaceae and Brassicaceae families, suggesting a common origin of these organs in sister lineages, both regulated by genes of the MADS domain (Carey *et al*. 2023). Consequently, *C. violacea* emerges as a promising model for studying nectary development because of its intrinsic traits and close relationship to *A. thaliana*.

The floral morphology of Cleomaceae is quite diverse, being mostly zygomorphic in symmetry. The floral monosymmetry that arises from the upward curvature of the spirals of the corolla and androecium leads to large variations in the shape of the nectary, as well as differences in colour, size and shape between the adaxial and abaxial petals (Patchell *et al*. 2011). Regarding the number of stamens, most genera of Cleomaceae have about six stamens (e.g., *Cleomella*, *Cleoserrata*, *Gynandropsis*, *Physostemon*, *Tarenaya*), but some more basal groups can have 35 to 250 stamens (*Kersia* and *Corynandra*, respectively) (Bayat *et al*. 2018).

While a trend towards stamen number reduction is evident within the Cleomaceae family, mirroring a broader pattern observed across angiosperms, exceptions exist, particularly within the Old‐World group. For example, *Cleome viscosa* (10–20 stamens), *C. karachiriensis* (6–8 stamens), and *C. brachycarpa* (6 stamens) have a higher number of stamens than typically seen in more derived species (Riaz *et al*. 2019). While stamen number does not provide a clear synapomorphy for the Cleomaceae family due to variations observed across species, the family's floral diversity, particularly in nectary characteristics, offers a unique opportunity to investigate the genetic controls underlying floral morphology and pollination strategies. The diversity within Cleomaceae provides a unique opportunity to explore unresolved questions about genetic control over floral nectary characteristics, enabling future research on crucial factors like floral morphology and pollination (Carey *et al*. 2023).

## FLORAL DIVERSITY IN CLEOMACEAE

**Fig. 1:**
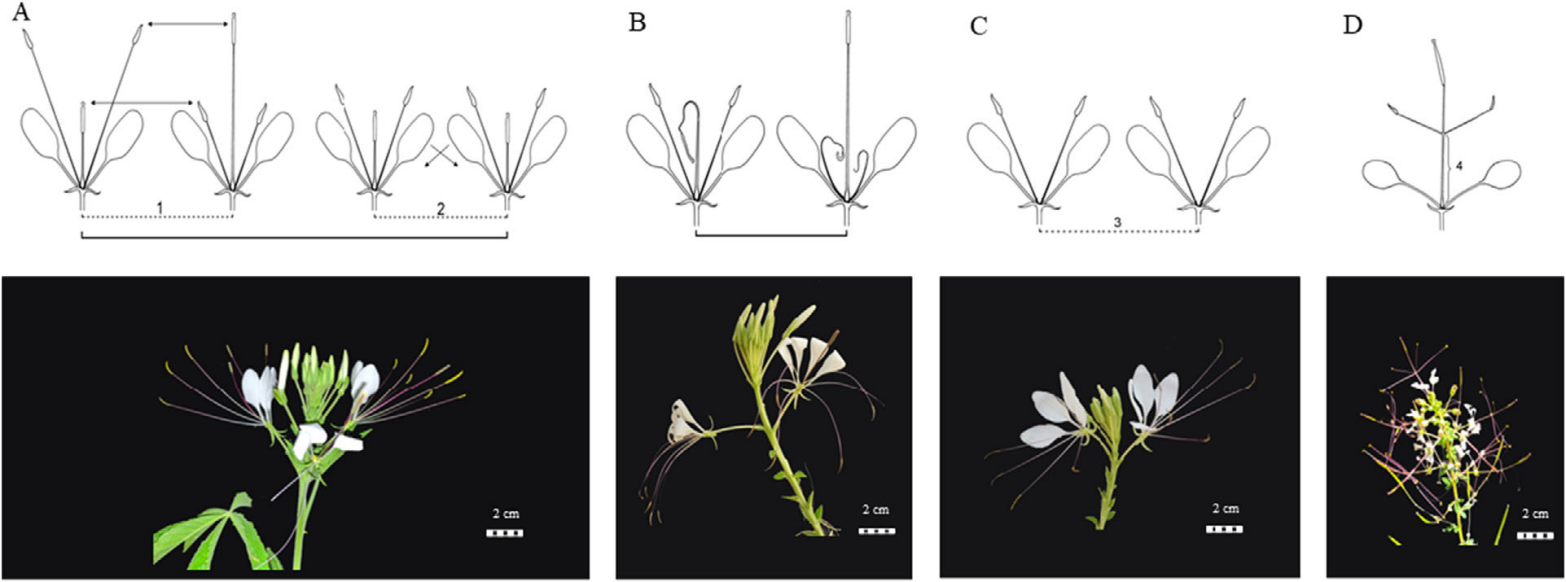
Strategies to promote cross‐pollination. (A) Hercogamy in *T. longicarpa* flowers: a physical barrier where the difference between the height of sexual organs in the flowers facilitates cross‐pollination. It can be of the dystilic type (1), with flowers of different morphology on the same plant; or homostilic (2), with flowers of the same morphology on the same plant. (B) Dichogamy in *T. longicarpa* flowers: a temporal barrier observed in hermaphrodite flowers, where the sexual organs mature at separate times, forcing cross‐pollination. (C) presence of flowers of only one sex on the same plant of *T. longicarpa* species, for example, bunches with only male flowers, forcing cross‐pollination (3). (D) elongation of the androgynophore in *G. gynandra* (4), where elongation of this structure raises the sexual organs of the flowers, facilitating the action of pollinating agents. Bars = 2 cm.

The diverse floral morphology within Cleomaceae facilitates a range of pollination strategies, from spontaneous self‐pollination to biotic cross‐pollination, the latter often accompanied by the production of copious amounts of nectar and pollen to attract pollinators. Some species of the family have nocturnal anthesis and are pollinated by bats and moths (e.g., *T. spinosa* (Machado *et al*. 2006)), others are pollinated by bees and butterflies (Cane 2008; Zohoungbogbo *et al*. 2018). In addition, other mechanisms like hercogamy, physical barriers (Fig. 1A; e.g., *G. gynandra*; Zohoungbogbo *et al*. 2018), dichogamy, temporal barriers (Fig. 1B) (e.g. *T. hassleriana*; Parma *et al*. 2023), are present in hermaphrodite species as an escape from self‐pollination, leading to increased genetic variability. The hercogamy – dysthilia – probably appeared more than once in Cleomaceae, since some groups do not have dystyly (e.g., *Cleome brachycarpa*, *C. violacea* and *C. karachiensis* have homostylia; Riaz *et al*. 2019). There are few studies on the Cleoamceae family, mostly concentrated on African and North American species, which explore reproductive biology and floral morphology (Cane 2008; Omondi *et al*. 2017; Zohoungbogbo *et al*. 2018). These regions have accessions that are particularly different from those found in the northeast of Brazil (Machado *et al*. 2006), and in other Brazilian regions (Parma *et al*. 2023).

The diversity observed in Cleomaceae could lead to a better understanding of pollination context and floral morphology. The reproductive success of angiosperms relies significantly on effective pollination strategies. Despite the recognized importance in the pollination of various *Passiflora* species, the genetic and developmental mechanisms of androgynophores remain poorly understood (Scorza & Dornelas 2014; Rocha *et al*. 2015). The distinct radial symmetry of the androgynophore in *G. gynandra* offers a notable mechanism for increasing interactions between pollinators and reproductive organs (Fig. 1D), attributed to rapid cellular elongation (Zenchyzen *et al*. 2024). Further research is needed to understand factors influencing androgynophore formation and investigate potential gene co‐option mechanisms in its development.

Importantly, the mass decline of pollinators is a global concern, with increasing evidence that pollinators are in crisis (Novais *et al*. 2016; Hallmann *et al*. 2017; Marshman *et al*. 2019). Cleomaceae have the capacity to clarify studies involving the development of varieties and hybrids with improved floral characteristics that reduce dependence on pollinators for production of nectar or pollen. In this sense, studies with MADS‐domain transcription factor genes allow us to find the transcription factors (TFs) responsible for diversity in floral morphology in Cleomaceae, as well as to understand the different types (sexes) of flowers.

It was demonstrated that *T. hassleriana* (Cleomaceae) has two copies of the floral B‐class gene PI, one similar to that found in other angiosperms, while the other is more related to that observed in Brassicaceae members (De Bruijn *et al*. 2018). In addition to differences between families, it is also possible to observe differences in the number of copies of some genes, as well as their expression within the same family. *T. hassleriana* has two highly similar AP3 paralogs that originated from a recent tandem duplication, while *G. gynandra* does not present this tandem duplication. However, to understand the dystyly observed in the family, much progress is still lacking. There is evidence of supergene control of the morphological and physiological components of heterostyly, but virtually nothing is known (Barrett 2002). Thus, understanding the factors behind the floral diversity will allow us to promote species for cross‐pollination or even to avoid it (self‐pollination).

Accordingly, the creation of cultivars with specialized nectar or nectar‐related attributes, with the aim of attracting and retaining pollinating insects, has emerged as a promising approach to meet both agricultural demands and pollinator conservation (Prasifka *et al*. 2018). An in‐depth study on diverse types of pollination and their effects on fruit set in Brazilian species of Cleomaceae conducted by Parma *et al*. (2023) reported cross‐pollination had the highest seed yield, followed by open pollination, which emphasizes the importance of pollinators mediating gene flow among individuals. On the other hand, strategies to avoid self‐fertilization found in most species (hercogamy or dichogamy) are strong reasons that might account for self‐pollination for lowest seed yield. Notably, several types of mating lacking pollen limitation are allowed once the species is proved to be self‐compatible.

Ultimately, Cleomaceae emerges as a promising group for improving crop yields through both cross‐pollination and self‐pollination strategies. The family offers valuable resources for both diurnal and nocturnal pollinators, attracting both managed and wild species through its exquisite floral features. Despite its selection for self‐fertility, which lowers its dependence on biotic pollinators, Cleomaceae remains highly attractive to a diverse array of pollinators, establishing it as a rich genetic reservoir for flowering and pollination traits (Zohoungbogbo *et al*. 2018).

## THE GENETIC BASIS OF CLEOMACEAE DIVERSITY

Although diversification of the Cleomaceae has been extensively addressed through molecular data (Hall 2008; Patchell *et al*. 2014), other characteristics (e.g., micromorphological, physiological, and genetic) have received less attention. Among the available approaches, cytological data has emerged as a promising source of evolutionary and taxonomic information, as it has the potential to offer insights into reproductive dynamics in various groups of angiosperms (Levin 2002; Sánchez‐Acebo 2005). However, cytological reports, ploidy number, and karyotype variation in Cleomaceae taxa are scarce. Recently, the chromosomal genome assembly of *G. gynandra* was presented (Zhao *et al*. 2022; Hoang *et al*. 2023), with 2C values ranging from 2.31–2.45 pg (Omondi *et al*. 2017; Hoang *et al*. 2023). Similarly, genome sizes of Cleomaceae species in Brazil have been determined, mainly for *Tarenaya* species (*T. hassleriana* 2C = 0.55 pg., *T. diffusa* 2C = 0.59 pg., *T. aculeata* and *T. microcarpa* 2C = 0.66 pg., *T. siliculifera* 2C = 0.77, and *T. spinosa*, *T. longicarpa*, *T. rosea*, *T. parviflora* 2C = 1.30 pg.), *Cleoserrata paludosa* 2C = 1.08, and *G. gynandra* 2C = 2.20 (Parma *et al*. 2022).

The plastome of some Cleomaceae species was recently characterized as resembling *G. gynandra*, with a size of 158.152 bp, harbouring a total of 131 genes including 37 tRNA genes, 87 protein‐coding genes, and seven rRNA genes (Shi & Bloom 2021); *C. paradoxa* with 159.393 bp, contains 133 genes, 29 of which encode tRNA, 81 encode proteins, and four are rRNA genes (ALJuhani & Aljohani 2022); *C. chrysantha* plastome is 158.111 bp, containing 136 genes, including 80 protein‐coding genes, 31 tRNA genes, and four rRNA genes (Alzahrani *et al*. 2021).

The chromosome number of Cleomaceae species has long been determined (Ammal, 1933; Olaoluwa & Azeez 2022; Subramanian & Susheela 1988). However, there are few specific studies in the family; some available data are from more comprehensive studies, such as characterization of angiosperms or Capparidaceae (the family to which Cleomaceae was formerly inserted). Additionally, studies that focused on *G. gynandra* revealed it as diploid, with 2*x* = 34 (Hoang *et al*. 2023), studies in Nigerian accessions determined *C. viscosa* and *C. rutidosperma as* 2*x* = 20 (Olaoluwa & Azeez 2022). It appears there is a tendency for most species in the family to be diploid, with some tetraploid groups (*C. chellidonii*) and in *Tarenaya* species; Inda *et al*. (2008). The chromosome number tends to be higher in the most basal species groups (Old World), reaching *x* = 29 in *Podandrogyne macrophylla*, *C. pilosa*, *C. stylosa*, *C. lechleri*, *C. anomala* and *C. moritziana* (Inda *et al*. 2008). Other groups have an intermediate chromosome number to most species in the New World group, such as *C. multicalis* (*x* = 20) (Inda *et al*. 2008), *x* = 16, 17 *C. chelidonii*, *Cleomella serrulata*, *Cleomella lutea* and *Melidiscus giganteus*. For the most derived species, with some exceptions from Old World groups, the chromosome number varies from 8 to 12, among these species, are *C. monophylla*, *C. tenella*, *C. felina*, *C. aspera*, *C. viscosa*, *C. cordobensis*, *C. stenophylla*, *C. rutidosperma*, *C. violacea*, *Polanisia dodecandra*, *T. crenopetala*, *T. diffusa*, *T. microcarpa*, *T. boliviensis*, *T. hassleriana*, *T. longicarpa*, *T. werdemanii*, *T. titubans*, *T. spinosa*, *T. pernambucensis*, *T. parviflora*, *T. torticarpa*, *T. chapalensis*, *T. rosea*, *T. tucumanensis*, and *T. aculeata* (Inda *et al*. 2008).

Studies on genome size, chromosome number, and ploidy events in Cleomaceae species are limited but are of foremost importance for understanding genetics of these species. A recent study of Cleomaceae accessions collected in Brazil revealed genome size variation that does not align with phylogenetic relationships, suggesting the occurrence of multiple polyploidy events within the genus (Parma *et al*. 2022). In this study, *G. gynandra* was identified as the sole C_4_ species among the analysed accessions, with a comparatively large nuclear genome. In contrast, the C_3_ species had both intra‐ and interspecific variation in genome size (Parma *et al*. 2022). This highlights the relationship between genetic information and species establishment, expanding our knowledge of the history of polyploidy, gene duplication and retention, and how these processes may have impacted the evolution of C_4_ photosynthesis in Cleomaceae.

## CLEOMACEAE AND EVOLUTION OF C_4_ PHOTOSYNTHESIS

The C_4_ photosynthetic mechanism has emerged independently more than 60 times in 19 different families (Sage 2016). C_4_ photosynthesis repeatedly and convergently evolved from the ancestral C_3_ state, most likely via C_2_ or C_3_‐C_4_ intermediate stages that would represent a transition between the two mechanisms (C_3_ and C_4_). In addition to the intermediate C_2_ mechanism, a range of intermediate phenotypes represent transitional states between the C_3_ and C_4_ mechanisms (Sage 2021). It is proposed that necessary steps for the development of C_4_ photosynthesis are (i) genetic preconditioning, (ii) anatomical preconditioning, (iii) enhancement of bundle sheath organelles, (iv) establishment of a photorespiratory CO_2_ pump based on confinement of glycine decarboxylase activity to bundle sheath cells, (v) enhancement of phosphoenolpyruvate carboxylase activity, (vi) establishment of C_4_ cycle, and (vii) optimization of the C_4_ syndrome (Sage 2004; Heckman *et al*. 2013). In this sense, many efforts have been made to understand how this transition between mechanisms occurs, since C_4_ enables higher biomass production with less water use compared to C_3_ plants (Lundgren *et al*. 2014). The presence of C_4_ enzymes in C_3_ species suggests that they do not need to evolve *de novo*, which likely facilitated recurrent evolution of the C_4_ pathway (Schuler *et al*. 2016; Reyna‐Llorens & Hibberd 2017).

The Cleomaceae family contains representatives with three different photosynthetic mechanisms, which together with phylogenetic proximity to Brassicaceae, makes it an interesting model family to understand important steps to acquire these characteristics (Bayat *et al*. 2018). Noteworthy, in addition to being family sisters, Brassicaceae and Cleomaceae genomes have significant synteny congruency (Schranz & Mitchell‐Olds 2006). Within the last two decades, studies to investigate important steps for C_4_ establishment using Cleomaceae members have been conducted. However, to date, only 15 species have been physiologically characterized (Marshall *et al*. 2007; Voznesenskaya *et al*. 2007; Reeves *et al*. 2018; Parma *et al*. 2022). Initial studies on the Cleomaceae family identified *G. gynandra* as a C_4_ species (Krenzer *et al*. 1975; Sankhla *et al*. 1975; Imbamba & Tieszen 1977; Raghavendra & Das 1978).

Subsequently, another study confirmed this and also identified *Sieruela monophylla*, *Cleome aspera*, and *C. viscosa* as C_3_ species (Rajendrudu and Rama Das, 1982). Further studies identified types of photosynthetic mechanism in Cleomaceae species (Marshall *et al*. 2007; Voznesenskaya *et al*. 2007; Feodorova *et al*. 2010; Reeves *et al*. 2018; Parma *et al*. 2022). In this scenario, some species and different accessions of the family were described physiologically (Table 1), in which three species were characterized as presenting C_4_ photosynthetic metabolism (*Areocleome oxalidea*, *Coalisina angustifolia* and *G. gynandra*), one with intermediate C_3_‐C_4_ metabolism (*Coalisina paradoxa*), and all others being C_3_. Notably, C_3_ species flexibility was identified, with the development of C_3_–C_4_ photosynthesis (Marshall *et al*. 2007) displaying genomic, ecological, and anatomical factors that facilitate acquisition of novel traits (Marshall *et al*. 2007; McKown & Dengler 2007; Christin *et al*. 2011, 2012, 2013). Furthermore, studies on nine accessions of *G. gynandra* demonstrated natural variation in key traits associated with C_4_ photosynthesis (Reeves *et al*. 2018). Therefore, the natural variation observed in *G. gynandra* can help to delineate genomic regions associated with C_4_ trait flexibility.

**Table 1:** Physiological variation in photosynthetic gas exchange and anatomical parameters among diverse species of Cleomaceae.

Species^a^	Mechanism	CO_2_ Г (μmol mol^−1^)	Photosynthetic rate (μmol m^−2^ s^−1^)	δ^13^C (‰)	Vein density (mm mm^−2^)	References
*Areocleome oxalídea*	C_4_	‐	‐	−14.1–15.8	‐	Koteyeva *et al*. (2011); Marshall *et al*. (2007); Voznesenskaya *et al*. (2007)
*Cleome africana*	C_3_	52.4–65.0	‐	−30.1	10	Koteyeva *et al*. (2011); Marshall *et al*. (2007); Voznesenskaya *et al*. (2007)
*Peritoma arborea*	C_3_	49.0	‐	−25.2–27.5	7	Marshall *et al*. (2007)
*Cleome marshallii* ^b^	C_3_	52.0	‐		7	Marshall *et al*. (2007)
*Cleome ornithodioides*	C_3_	66.5	‐	−28.0–29.7	‐	Voznesenskaya *et al*. (2007)
*Cleome violacea*	C_3_	51.0	‐	−29.5	8	Marshall *et al*. (2007)
*Coalisina angustifolia*	C_4_	‐	‐	−12.5–13.2	‐	Koteyeva *et al*. (2011); Marshall *et al*. (2007); Voznesenskaya *et al*. (2007)
*Coalisina paradoxa*	C_3_–C_4_	27.5	20	−23.8–26.0	11	Koteyeva *et al*. (2011); Marshall *et al*. (2007); Voznesenskaya *et al*. (2007)
*Gynandropsis gynandra*	C_4_	2.0–9.0	23–35	−13.9–17.1	6–10	Feodorova *et al*. (2010); Marshall *et al*. (2007); Reeves *et al*. (2018); Voznesenskaya *et al*. (2007)
*Kersia foliosa*	C_3_	40.0	‐	−22.8–25.6	8	Marshall *et al*. (2007)
*Melidiscus giganteus*	C_3_	66.9	‐	−23.4–29.3	‐	Voznesenskaya *et al*. (2007)
*Sieruela hirta*	C_3_	123.0	‐	−25.7–28.8	7	Marshall *et al*. (2007)
*Sieruela monophylla*	C_3_	62.0	23	−27.8	‐	Voznesenskaya *et al*. (2007)
*Tarenaya hassleriana*	C_3_	62.5	‐	−24.6–29.8	‐	Marshall *et al*. (2007)
*Tarenaya spinosa*	C_3_	50.0–66.7	‐	−26.41–30.6	5	Marshall *et al*. (2007); Voznesenskaya *et al*. (2007)

a All names have been updated according to IPNI 2020.

b Invalid name.

The C_4_ mechanism identified in Cleomaceae species has different origins, as evidenced by both molecular phylogeny (Patchell *et al*. 2014) and distinct types of Kranz anatomy (*A. oxalidea* and *G. gynandra* have atriplicoid and *C. angustifolia*, angustifolioid Kranz anatomy; Koteyeva *et al*. 2011). However, all three of these C_4_ species belong to the NAD‐dependent malic enzyme (NAD‐ME) subtype (Koteyeva *et al*. 2011). Interestingly, age‐dependent plasticity in decarboxylation mode was observed, with a mixed NAD‐ME and PEPCK types found in older leaves (Sommer *et al*. 2012). Notably, carbon isotope discrimination can differentiate species with C_3_ and C_4_ photosynthetic mechanisms (Voznesenskaya *et al*. 2007). However, C_3_‐C_4_ intermediates typically cannot be identified by carbon discrimination. Instead, gas exchange and metabolic analyses are needed to differentiate species with intermediate C_3_‐C_4_ (Schlüter & Weber 2016).

In an effort to characterize the species with regard to photosynthetic mechanism, isotopic carbon discrimination of 238 leaf samples (about 150 species) was used (Voznesenskaya *et al*. 2007). The results revealed a range of isotopic values, from −12.3‰ to −33.9‰. This suggests the presence of three centers of origin for C_4_ photosynthesis within the family (*Tarenaya siliculifera*, *Sieruela allamanii* and *S. gallaensis*). However, research on leaf anatomy and type of photosynthesis (using gas exchange) among members of the Cleomaceae remains limited (Marshall *et al*. 2007; Voznesenskaya *et al*. 2007).

Thus far, fundamental research into the Cleomaceae family, associated with physiological aspects, has laid the groundwork for furture studies, such as comparative genomics (Cheng *et al*. 2013; Külahoglu *et al*. 2014; Van Den Bergh *et al*. 2014; Williams *et al*. 2016). Regarding this, when comparing *G. gynandra* (C_4_) and *T. hassleriana* (C_3_), it was found that both species share the Th‐a WGD event (Van Den Bergh *et al*. 2014). A recent genome sequencing of *G. gynandra* alongside a comparative study with *T. hassleriana* revealed that, contrary to previous studies (Van Den Bergh *et al*. 2014; Bayat *et al*. 2018), there is a difference in the number of retained gene copies between these species (Zhao *et al*. 2022; Hoang *et al*. 2023). Indeed, genome duplication occurred in the common ancestor, followed by different patterns of loss and retention, resulting in the preservation of multiple copies of 29 genes associated with the C_4_ NAD‐ME photosynthetic subtype in *G. gynandra*, but not in *T. hassleriana*. These copies stem from both polyploidy and individual gene duplications (Zhao *et al*. 2022; Hoang *et al*. 2023). These distinct duplications, coupled with selective retention, recruitment, and modifications in gene expression, have driven evolution of the C_4_ cycle in *G. gynandra* (Zhao *et al*. 2022; Hoang *et al*. 2023; Huang *et al*. 2023). That precious resource places *G. gynandra* as a promising C_4_ model species, paving the way for functional and evolutionary comparative studies of C_3_ and C_4_ photosynthesis.

Despite advances in the field, mechanisms underlying the development of C_4_ anatomical features, compartmentalization of enzymes to specific cells, and specific patterns of differential gene expression that underpin the transition to C_4_ photosynthesis in the Cleomaceae (Bayat *et al*. 2018) remain to be fully elucidated. This dearth of knowledge can be attributed, at least in part, to the comparison of species with divergent evolutionary trajectories, such as *G. gynandra* (Old World) and *T. hassleriana* (New World), which belong to different clusters (Van Den Bergh *et al*. 2014; Bayat *et al*. 2018; Reeves *et al*. 2018; Hoang *et al*. 2023). Hence, it would be more appropriate to compare species of the same cluster, such as the genus *Coalisina*, which includes species with different photosynthetic mechanisms (C_3_, C_3_‐C_4_, and C_4_). Furthermore, analysis of a range of *G. gynandra* accessions can facilitate identification of genetic variations that underpin natural variation in C_4_ photosynthesis, which may inform strategies for crop improvement (Van Den Bergh *et al*. 2014; Reeves *et al*. 2018; Huang *et al*. 2021; Hoang *et al*. 2023). Consequently, as demonstrated above, the majority of knowledge concerning evolution of the C_4_ mechanism is derived from studies of other plants, including *Flaveria* and *Moricandia* (Rawsthorne *et al*. 1988; Schlüter & Weber 2016). However, within the Cleomaceae family, such studies remain limited.

Several studies have focused on the limited understanding of the differential anatomy and interference from external factors, such as water and light, in the C_4_ species *G. gynandra*. Recent studies focused on stomatal responses of *G. gynandra* and the potential for leveraging its intrinsic characteristics to enhance photosynthesis in existing C_4_ crops and support efforts for development of C_4_ photosynthesis in C_3_ crop species (Silva‐Alvim *et al.*, 2024). The results of Bernardo *et al*. (2023) highlighted distinct activation properties of *G. gynandra* K^+^ channels. The rapid stomatal opening promotes CO_2_ assimilation, while stomatal closure enhances WUE. These characteristics are independent of stomata morphology and density and may include the dynamics of K^+^ channels and the differential responsiveness of stomata to light quality and availability (Silva‐Alvim *et al.*, 2024; Bernardo *et al*. 2023).

Another intriguing feature that has recently been examined is the C_4_ plasmodesmal connectivity between bundle sheath and mesophyll cells, which facilitate enhanced metabolite flow efficiency between these cells. In *G. gynandra*, there is a higher frequency of plasmodesmata than in *A. thaliana*, which was found to be light induced (Schreier *et al*. 2024). Further research is necessary to ascertain whether this characteristic is a universal prerequisite for C_4_ photosynthesis in eudicots. *G. gynandra* has a significantly higher number of differentially expressed genes during de‐etiolation compared to *Arabidopsis*. Furthermore, studies have revealed that C_4_ species exhibit alterations in chromatin accessibility for specific C_4_ genes, resulting in augmented gene expression responses to light (Singh *et al*. 2023).

The environment exerts significant selection pressure on evolution of C_4_ species, primarily related to factors such as water availability, light intensity, and high temperatures (Keeley & Rundel 2003; Osborne & Sack 2012; Zhou *et al*., 2018). These factors directly influence the survival and reproductive success of C_3_ species, which may have led to selection of adaptive characteristics, such as those found in C_4_ species.

In the Cleomaceae family, we highlight *G. gynandra*, a diploid C_4_ eudicotyledonous species, with a large genome, whose C_4_ genes may be under positive selection pressure. Recent studies highlight the potential of *G. gynandra* as a model system to elucidate the evolutionary C_4_ pathway within the Cleomaceae family, especially in light of their capacity for genetic manipulation (Hoang *et al*. 2023). The combination of these characteristics confers substantial advantages on the eudicotyledonous species compared to other C_4_ species currently used as models to insert desirable characteristics of C_4_ plants into economically important C_3_ species, such as rice (*Oryza sativa*). Consequently, this enhances the family's potential to generate *de novo* C_4_‐engineered crops.

## REGULATION OF C_4_ GENE EXPRESSION

The evolution of C_4_ photosynthesis relied on regulatory rewiring rather than protein sequence changes. Across multiple lineages, novel *cis*‐regulatory elements in promoters and untranslated regions established cell‐specific expression patterns (Gowik & Westhoff 2011; Mendieta *et al*. 2024; Swift *et al*. 2024). In *Flaveria*, the C_4_‐PEPC promoter acquired a conserved enhancer (MEM1) and retrotransposon insertions, conferring mesophyll‐specific expression (Gowik & Westhoff 2011; Lyu *et al*. 2025). Similarly, single‐cell chromatin profiling and comparative genomics in grasses revealed recruitment of cell‐type‐specific enhancers and transcription factor binding sites, often derived from antient mobile elements, which drive mesophyll and bundle‐sheath partitioning (Mendieta *et al*. 2024; Chen *et al*. 2025).

The above findings strongly imply the co‐option of pre‐existing genes into newly evolved regulatory networks driving the C_3_ to C_4_ metabolism transition (Schlüter & Weber 2020). In Cleomaceae, shared whole‐genome duplication events generated redundant gene copies amenable to neofunctionalization (Van Den Bergh *et al*. 2014; Huang *et al*. 2021; Hoang *et al*. 2023). In Brassicaceae, GLDP1 coding for the P‐subunit of the glycine decarboxylase complex engaged in photorespiration, acquired BS‐specific expression by a promoter retrotransposon insertion, enabling a C_2_‐like shuttle during early C_4_ evolution (Triesch *et al*. 2024). In *Moricandia* (a C2 species), BS cells already upregulate portions of the C4 metabolic and photorespiratory cycle (Triesch *et al*. 2025), suggesting partial deployment of the C_4_ regulatory program. Regulatory elements appear to have been independently co‐opted in different lineages, while in Cleomaceae, multiple C_4_ genes share common promoter “gateways” (Williams *et al*. 2016), suggesting convergent recruitment of transcriptional modules.

Cleomaceae genomic resources are still underrepresented, which limits insights into the rewiring of *cis*‐regulatory elements, chromatin states, and transcription regulatory networks driving C_4_ evolution emergence in this Brassicaceae sister family. High‐quality genome assemblies spanning C_3_, intermediate and C_4_ lineages are urgently needed to reconstruct the regulatory routes to C_4_ metabolism and to distinguish conserved mechanisms from lineage‐specific mechanisms.

## IMPORTANCE AND POTENTIAL OF THE CLEOMACEAE FAMILY: NUTRITIONAL, MEDICINAL, AND AGRONOMIC ASPECTS

The Cleomaceae family comprises species that are of socio‐economic, medicinal, and ornamental importance (Anburaj *et al*. 2011; Rathore *et al*. 2013; Singh *et al*. 2018). The species *G. gynandra* is important in nutrition in arid regions in sub‐Saharan Africa and Asia and is regarded as a species of considerable socio‐economic importance linked to food security (Wu *et al*. 2018). *G. gynandra* possesses the potential to enhance food security and address micronutrient deficiencies (Omondi *et al*. 2017). Beyond its notable vitamin A and C content (Uusiku *et al*. 2010), *G. gynandra* leaves are a rich source of beneficial polyphenolic phytochemicals, including flavonoids and polyphenols (Chataika *et al*. 2022), which have been shown to have significant health benefits. Furthermore, the presence of violaxanthin, α, β, and γ‐tocopherol, luteolin, ascorbic acid, α and β‐carotene, and, β‐cryptoxanthin has also been documented (Chand *et al*. 2022).

Leaves of *G. gynandra* have high concentrations of vitamin C and nutrients such as calcium, phosphorus, and iron (Houdegbe *et al*. 2018; Moyo *et al*. 2018). It is noteworthy that these nutrients are even more abundant in *G. gynandra* than in commercial vegetable leaves, such as *Brassica oleracea* and *Beta vulgaris* (Moyo *et al*. 2018). Research of the nutritional composition of other species within the family and their possible benefits to human and animal health is currently limited. However, only *G. gynandra* has been thoroughly characterized from the nutritional point of view.

Some species, including *T. aculeta*, *T. longicarpa*, *Cleome rutidosperma* (Okonwu *et al*. 2017), and *C. viscosa*, have been used in drug treatments for various illnesses (Singh *et al*. 2018). In addition, many species produce essential oils that have antifungal, antimicrobial, and insecticidal activity. Consequently, extracts of certain Cleomaceae species have inhibitory effects on the growth of various organisms considered to be pathogenic in diverse cultural contexts. These extracts are also a valuable source of natural bioactive antioxidants. Noteworthy species include *C. droserifolia*, *C. trinervia* (Muhaidat *et al*. 2011), *C. rutidosperma* (McNeil *et al*. 2018), *C. brachycarpa* (Rassouli et al., 2014), and C. amblyocarpa (Shahin *et al*. 2018), *C. serrata* (McNeil *et al*. 2012), *C. heratensis* (Nasseri *et al*. 2017; Nasseri et al., 2019), *C. iberica* (Moridi Farimani *et al*. 2017), *G. gynandra* (Nyalala & Grout 2007), and *T. spinosa* (Da Silva *et al*. 2016; McNeil *et al*. 2010). In contrast, *T. hassleriana* and *T. spinosa* (Tsai *et al*. 2012) are regarded as ornamental species, extensively cultivated in gardens and botanical gardens worldwide.

Even though the family's importance is well recognized in several fields, research on productivity of this plant group remains scarce (Chweya & Mnzava 1997; Oluoch *et al*. 2009; Houdegbe *et al*. 2018). In this regard, the development of suitable agronomic practices for enhancing yield is imperative to expedite domestication of Cleomaceae species and promote large‐scale production (Houdegbe *et al*. 2018; Onyango *et al.*, 2013). Consequently, further research is required to investigate agronomic‐related issues, including planting density, fertilization, pest management, irrigation needs, transplanting time, and cutting modes to enhance yield (Houdegbe *et al*. 2018). To date, studies in this area have been conducted exclusively on *G. gynandra* (Chweya & Mnzava 1997; Oluoch *et al*. 2009; Ochuodho et al., 2012; Houdegbe *et al*. 2018). Houdegbe *et al*. (2018) confirmed a planting density of ca. 445.000 plants ha^−1^ (spacing of 15 cm × 15 cm), similarly, Chweya & Mnzava (1997), Ochuodho et al., (2012), Oluoch *et al*. (2009) found the average yield of ca. 30 t ha^−1^. Notably, the validated biomass production in *G. gynandra* is substantial, particularly when compared to other crops and vegetables, given its consumption as a food source. Consequently, awareness of the beneficial nutrient content in *G. gynandra* has the potential to increase its consumption, thereby leading to improved human health (Omondi *et al*. 2017).

In addition to studies on productivity, a significant knowledge gap exists in the field of germination research. It is known that some species exhibit dormancy, and that specific processes are required to enhance germination. For instance, in *G. gynandra* Zohoungbogbo *et al*. (2018) found a germination rate of only 25%, while Ekpong (2009) found only 17% germination without any prior treatment. Recent research has revealed that *G. gynandra* seeds can germinate under various conditions, covering a wide range of temperatures. These seeds exhibit both physical and physiological dormancy, which can be overcome through combined ageing and immersion in GA_3_ (Saifullah *et al*. 2023). It is recommended that a post‐maturation period of >6 months be implemented to enhance germination (Saifullah *et al*. 2023). For other species, such as *T. longicarpa* (De Castro *et al*. 2014), *Cleomella lutea* and *C. serrulata* (Cane 2008), *T. hassleriana* and *Polanisia dodecandra* (Gomez Rabo & Anderson 2010), seed dormancy was also found. While these studies enhanced germination rates of species (~90%) by extending the storage duration and using alternative temperatures, another study employing treatments including varying concentrations of GA_3_, KNO_3_, leaching, pre‐chilling, soaking, and pre‐heating at various temperatures, which elevated the germination rate of *G. gynandra* (Ekpong 2009). In general, seed germination within the Cleomaceae family poses significant challenges for many species, largely related to the presence of seed physical and physiological dormancy. Further research is necessary to enhance methodologies and extend their application to a broader range of species within the family. This will contribute to the efficient cultivation of Cleomaceae and expand their use in diverse contexts.

## CONCLUDING REMARKS

The Cleomaceae family presents a promising avenue for addressing long‐standing questions in plant research. Consequently, agricultural science offers significant potential for investigation across a range of disciplines, including evolution of C_4_ photosynthesis and enhancement of C_3_ species productivity in unfavourable conditions. Notably, the Cleomaceae family has a unique capacity for self‐pollination, which, when coupled with its use as a proxy for hybrid production, attracts a diverse array of pollinators. This relationship underscores the family's ecological significance, as it plays a crucial role in preservation and growth of its population through active management practices. Notably, this family harbours the potential for development of plant‐derived pharmaceuticals. Additionally, certain species are edible and can be utilized as vegetables with high nutritional value.

In conclusion, there are numerous avenues for advancing research in various fields using the Cleomaceae family. However, there is still a need for fundamental studies focusing on the existing diversity of species and genetic resources. Such fundamental studies are imperative for the preservation and protection of the family, the establishment of protocols for *in vitro* cultivation and transformation, the development of germplasm banks, and many other crucial endeavours.

## AUTHOR CONTRIBUTIONS

PFG, DFP, JSI and WEBB contributed equally to the conceptualization of the review, literature selection, drafting of the manuscript, and creation of figures and tables. APMW, WLA and ANN provided specialized input on all sections, critically reviewed the manuscript for intellectual content, and approved the final version for submission. All authors read and approved the final version.

## References

[plb70130-bib-0001] ALJuhani W.S. , Aljohani A.Y. (2022) Complete chloroplast genome of the medicinal plant *Cleome paradoxa* r.Br. Ex DC: comparative analysis, and phylogenetic relationships among the members of Cleomaceae. Gene, 845, 146851. 10.1016/j.gene.2022.146851 36057366

[plb70130-bib-0002] Al‐Shehbaz I.A. (1985) The genera of Thelypodieae (Cruciferae; Brassicaceae) in the southeastern United States. Journal of the Arnold Arboretum, 66, 95–111. 10.5962/bhl.part.13180

[plb70130-bib-0003] Alzahrani D. , Albokhari E. , Yaradua S. , Abba A. (2021) Complete chloroplast genome sequences of *Dipterygium glaucum* and *Cleome chrysantha* and other Cleomaceae species, comparative analysis and phylogenetic relationships. Saudi Journal of Biological Sciences, 28, 2476–2490. 10.1016/j.sjbs.2021.01.049 33911961 PMC8071925

[plb70130-bib-1006] Ammal J.E.K. (1933) The chromosome number of *Cleome viscosa* Linn. Current Science, 1, 328.

[plb70130-bib-0004] Anburaj J. , Singh C.R. , Sundarraj S. , Kannan S. (2011) In vitro regeneration of *Cleome viscosa*–an important medicinal herb. Journal of Cell Molecular Biology, 9, 37–44.

[plb70130-bib-0005] Barrett R.L. , Roalson E.H. , Ottewell K. , Byrne M. , Govindwar S.P. , Yadav S.R. , Tamboli A.S. , Gholave A.R. (2017) Resolving generic boundaries in Indian‐Australasian Cleomaceae: circumscription of *Areocleome*, *Arivela*, and *Corynandra* as distinct genera. Systematic Botany, 42, 694–708.

[plb70130-bib-0006] Barrett S.C.H. (2002) The evolution of plant sexual diversity. Nature Reviews. Genetics, 3, 274–284. 10.1038/nrg776 11967552

[plb70130-bib-0007] Bayat S. , Schranz M.E. , Roalson E.H. , Hall J.C. (2018) Lessons from Cleomaceae, the sister of crucifers. Trends in Plant Science, 23, 808–821. 10.1016/j.tplants.2018.06.010 30006074

[plb70130-bib-0008] Bernardo E.L. , Sales C.R.G. , Cubas L.A. , Vath R.L. , Kromdijk J. (2023) A comparison of stomatal conductance responses to blue and red light between C_3_ and C_4_ photosynthetic species in three phylogenetically‐controlled experiments. Frontiers in Plant Science, 14, 1253976. 10.3389/fpls.2023.1253976 37828928 PMC10565490

[plb70130-bib-0010] Cane J.H. (2008) Breeding biologies, seed production and species‐rich bee guilds of *Cleome lutea* and *Cleome serrulata* (Cleomaceae). Plant Species Biology, 23, 152–158. 10.1111/j.1442-1984.2008.00224.x

[plb70130-bib-0011] Carey S. , Zenchyzen B. , Deneka A.J. , Hall J.C. (2023) Nectary development in *Cleome violacea* . Frontiers in Plant Science, 13, 1085900. 10.3389/fpls.2022.1085900 36844906 PMC9949531

[plb70130-bib-0012] Castro T.C.D. , Simões‐Gurgel C. , Ribeiro I.G. , Coelho M.G.P. , Albarello N. (2014) Morphological aspects of fruits, seeds, seedlings and in vivo and in vitro germination of species of the genus cleome. Journal of Seed Science, 36, 326–335. 10.1590/2317-1545v36n31013

[plb70130-bib-0013] Chand J. , Panda S.R. , Jain S. , Murty U.S.N. , Das A.M. , Kumar G.J. , Naidu V.G.M. (2022) Phytochemistry and polypharmacology of cleome species: a comprehensive ethnopharmacological review of the medicinal plants. Journal of Ethnopharmacology, 282, 114600. 10.1016/j.jep.2021.114600 34487845

[plb70130-bib-0014] Chataika B.Y. , Akundabweni L.S.‐M. , Sibiya J. , Achigan‐Dako E.G. , Sogbohossou D.E.O. , Kwapata K. , Awala S. (2022) Major production constraints and spider plant [*Gynandropsis gynandra* (L.) Briq.] traits preferences amongst smallholder farmers of northern Namibia and Central Malawi. Frontiers in Sustainable Food Systems, 6, 831821. 10.3389/fsufs.2022.831821

[plb70130-bib-0015] Chen L. , Jia Y. , Zhou Z. , Jiang Q. , Liu J. , Gu J. , Chen C. , Cheng S. , Chu J. , Liu X. , Lin Y. , Li X. (2025) Genomic and cis‐regulatory basis of a plastic C_3_‐C_4_ photosynthesis in *Eleocharis baldwinii* . Advanced Science, 12(32), e15681. 10.1002/advs.202415681 40444461 PMC12407342

[plb70130-bib-0016] Cheng S. , Van Den Bergh E. , Zeng P. , Zhong X. , Xu J. , Liu X. , Hofberger J. , De Bruijn S. , Bhide A.S. , Kuelahoglu C. , Bian C. , Chen J. , Fan G. , Kaufmann K. , Hall J.C. , Becker A. , Bräutigam A. , Weber A.P.M. , Shi C. , Zheng Z. , Li W. , Lv M. , Tao Y. , Wang J. , Zou H. , Quan Z. , Hibberd J.M. , Zhang G. , Zhu X.‐G. , Xu X. , Schranz M.E. (2013) The *Tarenaya hassleriana* genome provides insight into reproductive trait and genome evolution of crucifers. The Plant Cell, 25, 2813–2830. 10.1105/tpc.113.113480 23983221 PMC3784582

[plb70130-bib-0017] Christin P.‐A. , Besnard G. , Edwards E.J. , Salamin N. (2012) Effect of genetic convergence on phylogenetic inference. Molecular Phylogenetics and Evolution, 62, 921–927. 10.1016/j.ympev.2011.12.002 22197805

[plb70130-bib-0018] Christin P.‐A. , Osborne C.P. , Chatelet D.S. , Columbus J.T. , Besnard G. , Hodkinson T.R. , Garrison L.M. , Vorontsova M.S. , Edwards E.J. (2013) Anatomical enablers and the evolution of C_4_ photosynthesis in grasses. National Academy of Sciences of the United States of America, 110, 1381–1386. 10.1073/pnas.1216777110 PMC3557070 23267116

[plb70130-bib-0019] Christin P.‐A. , Sage T.L. , Edwards E.J. , Ogburn R.M. , Khoshravesh R. , Sage R.F. (2011) Complex evolutionary transitions and the significance of C_3_‐C_4_ intermediate forms of photosynthesis in Molluginaceae: evolution of C_4_ photosynthesis in Molluginaceae. Evolution, 65, 643–660. 10.1111/j.1558-5646.2010.01168.x 20955197

[plb70130-bib-0020] Chweya J.A. , Mnzava N.A. (1997) Cat's whiskers, cleome Gynandra L. Bioversity International, Rome, Italy.

[plb70130-bib-0021] Costa‐e‐Silva M. (2000) O gênero Cleome L. (*Capparaceae Juss*.) para o Brasil. Unpublished Ph D Disser‐Tation, Universidade Federal Rural de Pernambuco, Recife, Brazil.

[plb70130-bib-0022] De Bruijn S. , Zhao T. , Muiño J.M. , Schranz E.M. , Angenent G.C. , Kaufmann K. (2018) PISTILLATA paralogs in *Tarenaya hassleriana* have diverged in interaction specificity. BMC Plant Biology, 18, 368. 10.1186/s12870-018-1574-0 30577806 PMC6303913

[plb70130-bib-1002] Eichler A.W. (1865) Capparideae. In: von Martius C.F.P. (Ed), Flora brasiliensis, Vol. 13. Frid Fleischer, München, pp 237–292. Disponível em: https://www.biodiversitylibrary.org/page/13807

[plb70130-bib-0025] Ekpong B. (2009) Effects of seed maturity, seed storage and pre‐germination treatments on seed germination of cleome (*Cleome gynandra* L.). Scientia Horticulturae, 119, 236–240. 10.1016/j.scienta.2008.08.003

[plb70130-bib-0027] Feodorova T.A. , Voznesenskaya E.V. , Edwards G.E. , Roalson E.H. (2010) Biogeographic patterns of diversification and the origins of C_4_ in *cleome* (Cleomaceae). Systematic Botany, 35, 811–826. 10.1600/036364410X539880

[plb70130-bib-0028] Gomez Rabo N.N. , Anderson N.O. (2010) Germination of *Cleome hassleriana* and *Polanisia dodecandra* seed lots in response to light, temperature and stratification. Research Journal of Seed Science., 3, 1–17. 10.3923/rjss.2010.1.17

[plb70130-bib-0029] Gowik U. , Westhoff P. (2011) The path from C_3_ to C_4_ photosynthesis. Plant Physiology, 155, 56–63. 10.1104/pp.110.165308 20940348 PMC3075750

[plb70130-bib-0030] Hall J.C. (2008) Systematics of Capparaceae and Cleomaceae: an evaluation of the generic delimitations of *Capparis* and *cleome* using plastid DNA sequence data. This paper is one of a selection of papers published in the Special Issue on Systematics Research. Botany, 86, 682–696. 10.1139/B08-026

[plb70130-bib-0031] Hall J.C. , Sytsma K.J. , Iltis H.H. (2002) Phylogeny of Capparaceae and Brassicaceae based on chloroplast sequence data. American Journal of Botany, 89, 1826–1842. 10.3732/ajb.89.11.1826 21665611

[plb70130-bib-0032] Hallmann C.A. , Sorg M. , Jongejans E. , Siepel H. , Hofland N. , Schwan H. , Stenmans W. , Müller A. , Sumser H. , Hörren T. , Goulson D. , De Kroon H. (2017) More than 75 percent decline over 27 years in total flying insect biomass in protected areas. PLoS One, 12, e0185809. 10.1371/journal.pone.0185809 29045418 PMC5646769

[plb70130-bib-0033] Heckman D. , Schulze S. , Denton A. , Gowik U. , Westhoff P. , Weber A.P.M. , Lercher M.J. (2013) Predicting C_4_ photosynthesis evolution: modular, individually adaptive steps on a Mount Fuji fitness landscape. Cell, 153, 1579–1588. 10.1016/j.cell.2013.04.058 23791184

[plb70130-bib-1003] Heilborn O. (1931) Section Fruticosae Eichl. of the genus *Cleome* L. Arkiv för Botanik, 23A, 1–19. Disponível em: https://rex.libraries.wsu.edu/view/pdfCoverPage?download=true&filePid=13351736580001842&instCode=01ALLIANCE_WSU

[plb70130-bib-0035] Hoang N.V. , Sogbohossou E.O.D. , Xiong W. , Simpson C.J.C. , Singh P. , Walden N. , Van Den Bergh E. , Becker F.F.M. , Li Z. , Zhu X.‐G. , Brautigam A. , Weber A.P.M. , Van Haarst J.C. , Schijlen E.G.W.M. , Hendre P.S. , Van Deynze A. , Achigan‐Dako E.G. , Hibberd J.M. , Schranz M.E. (2023) The *Gynandropsis gynandra* genome provides insights into whole‐genome duplications and the evolution of C_4_ photosynthesis in Cleomaceae. The Plant Cell, 35, 1334–1359. 10.1093/plcell/koad018 36691724 PMC10118270

[plb70130-bib-0038] Houdegbe C.A. , Sogbohossou E.O.D. , Achigan‐Dako E.G. (2018) Enhancing growth and leaf yield in *Gynandropsis gynandra* (L.) Briq. (Cleomaceae) using agronomic practices to accelerate crop domestication. Scientia Horticulturae, 233, 90–98. 10.1016/j.scienta.2018.01.035

[plb70130-bib-0039] Huang C.‐F. , Liu W.‐Y. , Lu M.‐Y.J. , Chen Y.‐H. , Ku M.S.B. , Li W.‐H. (2021) Whole‐genome duplication facilitated the evolution of C_4_ photosynthesis in *Gynandropsis gynandra* . Molecular Biology and Evolution, 38, 4715–4731. 10.1093/molbev/msab200 34191030 PMC8557433

[plb70130-bib-0040] Huang C.‐F. , Liu W.‐Y. , Yu C.‐P. , Wu S.‐H. , Ku M.S.B. , Li W.‐H. (2023) C_4_ leaf development and evolution. Current Opinion in Plant Biology, 76, 102454. 10.1016/j.pbi.2023.102454 37743123

[plb70130-bib-1004] Iltis H.H. (1952) A revision of the New World species of Cleome. Ph.D. Thesis, Washington University, St. Louis, MO, USA: 335 pp.

[plb70130-bib-1001] Iltis H.H. (1957) Studies in the Capparidaceae. III. Evolution and Phylogeny of the Western North American Cleomoideae. Annals of the Misosuri Botanical Garden, 44, 77–119. 10.2307/2394679

[plb70130-bib-0042] Iltis H.H. , Hall J.C. , Cochrane T.S. , Sytsma K.J. (2011) Studies in the Cleomaceae I. On the separate recognition of Capparaceae, Cleomaceae, and Brassicaceae^1^ . Annals of the Missouri Botanical Garden, 98, 28–36. 10.3417/2007017

[plb70130-bib-0043] Imbamba S.K. , Tieszen L.L. (1977) Influence of light and temperature on photosynthesis and transpiration in some C_3_ and C_4_ vegetable plants from Kenya. Physiologia Plantarum, 39, 311–316. 10.1111/j.1399-3054.1977.tb01890.x

[plb70130-bib-0044] Inda L.A. , Torrecilla P. , Catalán P. , Ruiz‐Zapata T. (2008) Phylogeny of *cleome L*. and ITS close relatives *Podandrogyne ducke* and *Polanisia Raf*. (Cleomoideae, Cleomaceae) based on analysis of nuclear ITS sequences and morphology. Plant Systematics and Evolution, 274, 111–126. 10.1007/s00606-008-0026-y

[plb70130-bib-0046] Keeley J.E. , Rundel P.W. (2003) Evolution of CAM and C_4_ carbon‐concentrating mechanisms. International Journal of Plant Sciences, 164, S55–S77. 10.1086/374192

[plb70130-bib-0047] Keller S. (1979) A revision of the genus Wislizenia (Capparidaceae) based on population studies. Brittonia, 31, 333–351.

[plb70130-bib-0048] Kers L. (2003) Capparaceae, Flowering plants dicotyledons: malvales, capparales and non‐betalain caryophyllales. Springer‐Verlag, Berlin and Heilderberg, pp 36–56.

[plb70130-bib-0049] Koteyeva N.K. , Voznesenskaya E.V. , Roalson E.H. , Edwards G.E. (2011) Diversity in forms of C_4_ in the genus cleome (Cleomaceae). Annals of Botany, 107, 269–283. 10.1093/aob/mcq239 21147832 PMC3025737

[plb70130-bib-0050] Krenzer E.G. , Moss D.N. , Crookston R.K. (1975) Carbon dioxide compensation points of flowering plants. Plant Physiology, 56, 194–206. 10.1104/pp.56.2.194 16659272 PMC541789

[plb70130-bib-0051] Külahoglu C. , Denton A.K. , Sommer M. , Maß J. , Schliesky S. , Wrobel T.J. , Berckmans B. , Gongora‐Castillo E. , Buell C.R. , Simon R. , De Veylder L. , Bräutigam A. , Weber A.P.M. (2014) Comparative transcriptome atlases reveal altered gene expression modules between two Cleomaceae C_3_ and C_4_ plant species. The Plant Cell., 26, 3243–3260. 10.1105/tpc.114.123752 25122153 PMC4371828

[plb70130-bib-0053] Levin D.A. (2002) The role of chromosomal change in plant evolution. Oxford University Press, USA.

[plb70130-bib-0054] Lundgren M.R. , Osborne C.P. , Christin P.‐A. (2014) Deconstructing Kranz anatomy to understand C_4_ evolution. Journal of Experimental Botany, 65, 3357–3369. 10.1093/jxb/eru186 24799561

[plb70130-bib-0055] Lyu M.‐J.A. , Du H. , Yao H. , Zhang Z. , Chen G. , Huang Y. , Ni X. , Chen F. , Zhao Y.‐Y. , Tang Q. , Miao F. , Wang Y. , Zhao Y. , Lu H. , Fang L. , Gao Q. , Qi Y. , Zhang Q. , Zhang J. , Yang T. , Cui X. , Liang C. , Lu T. , Zhu X.‐G. (2025) A dominant role of transcriptional regulation during the evolution of C_4_ photosynthesis in *Flaveria* species. Nature Communications, 16, 1643. 10.1038/s41467-025-56901-y PMC11828953 39952962

[plb70130-bib-0056] Machado I. , Cristina Lopes A. , Valentina Leite A. , Virgíniade Brito Neves C. (2006) *Cleome spinosa* (Capparaceae): polygamodioecy and pollination by bats in urban and caatinga areas, northeastern Brazil. Botanische Jahrbücher Für Systematik, Pflanzengeschichte und Pflanzengeographie, 127, 69–82. 10.1127/0006-8152/2006/0127-0069

[plb70130-bib-0057] Marshall D.M. , Muhaidat R. , Brown N.J. , Liu Z. , Stanley S. , Griffiths H. , Sage R.F. , Hibberd J.M. (2007) *Cleome*, a genus closely related to *Arabidopsis*, contains species spanning a developmental progression from C_3_ to C_4_ photosynthesis. The Plant Journal, 51, 886–896. 10.1111/j.1365-313X.2007.03188.x 17692080

[plb70130-bib-0058] Marshman J. , Blay‐Palmer A. , Landman K. (2019) Anthropocene crisis: climate change, pollinators, and food security. Environment, 6, 22. 10.3390/environments6020022

[plb70130-bib-0059] McKown A.D. , Dengler N.G. (2007) Key innovations in the evolution of Kranz anatomy and C_4_ vein pattern in *Flaveria* (Asteraceae). American Journal of Botany, 94, 382–399. 10.3732/ajb.94.3.382 21636408

[plb70130-bib-0060] McNeil M.J. , Porter R.B. , Williams L.A. , Rainford L. (2010) Chemical composition and antimicrobial activity of the essential oils from *Cleome spinosa* . Natural Product Communications, 5, 1934578X1000500833. 20839641

[plb70130-bib-0061] McNeil M.J. , Porter R.B.R. , Rainford L. , Dunbar O. , Francis S. , Laurieri N. , Delgoda R. (2018) Chemical composition and biological activities of the essential oil from *Cleome rutidosperma* DC. Fitoterapia, 129, 191–197. 10.1016/j.fitote.2018.07.006 29981873

[plb70130-bib-0062] McNeil M.J. , Porter R.B.R. , Williams L.A.D. (2012) Chemical composition and biological activity of the essential oil from Jamaican *Cleome serrata* . Natural Product Communications, 7, 1231–1232. 10.1177/1934578X1200700934 23074917

[plb70130-bib-0063] Mendieta J.P. , Tu X. , Jiang D. , Yan H. , Zhang X. , Marand A.P. , Zhong S. , Schmitz R.J. (2024) Investigating the cis‐regulatory basis of C_3_ and C_4_ photosynthesis in grasses at single‐cell resolution. Proceedings of the National Academy of Sciences, 121, e2402781121. 10.1073/pnas.2402781121 PMC11459142 39312655

[plb70130-bib-0064] Moridi Farimani M. , Nazarianpoor E. , Rustaie A. , Akhbari M. (2017) Phytochemical constituents and biological activities of *Cleome iberica* DC. Natural Product Research, 31, 1329–1332. 10.1080/14786419.2016.1239093 27731648

[plb70130-bib-0065] Moyo M. , Amoo S.O. , Aremu A.O. , Gruz J. , Šubrtová M. , Jarošová M. , Tarkowski P. , Doležal K. (2018) Determination of mineral constituents, phytochemicals and antioxidant qualities of *Cleome gynandra*, compared to *Brassica oleracea* and *Beta vulgaris* . Frontiers in Chemistry, 5, 128. 10.3389/fchem.2017.00128 29354633 PMC5758552

[plb70130-bib-0066] Muhaidat R. , Sage T.L. , Frohlich M.W. , Dengler N.G. , Sage R.F. (2011) Characterization of C_3_–C_4_ intermediate species in the genus *Heliotropium* L. (Boraginaceae): anatomy, ultrastructure and enzyme activity. Plant, Cell & Environment, 34, 1723–1736. 10.1111/j.1365-3040.2011.02367.x 21631534

[plb70130-bib-0067] Nasseri M.A. , Behravesh S. , Allahresani A. (2017) Essential oil composition of *Cleome heratensis* (Capparaceae) at different growing stages. Iranian Chemical Communication, 5, 364–371.

[plb70130-bib-1010] Nasseri M. .A. , Behravesh S. , Allahresani A. , Kazemnejadi M. (2019) Phytochemical and antioxidant studies of *Cleome heratensis* (Capparaceae) plant extracts. Bioresources and Bioprocessing, 6, Article 5. 10.1186/s40643-019-0240-1

[plb70130-bib-0068] Newell C.A. , Brown N.J. , Liu Z. , Pflug A. , Gowik U. , Westhoff P. , Hibberd J.M. (2010) Agrobacterium tumefaciens‐mediated transformation of *Cleome gynandra* L., a C_4_ dicotyledon that is closely related to *Arabidopsis thaliana* . Journal of Experimental Botany, 61, 1311–1319. 10.1093/jxb/erq009 20150516 PMC2837259

[plb70130-bib-0069] Novais S.M.A. , Nunes C.A. , Santos N.B. , D'Amico A.R. , Fernandes G.W. , Quesada M. , Braga R.F. , Neves A.C.O. (2016) Effects of a possible pollinator crisis on food crop production in Brazil. PLoS One, 11, e0167292. 10.1371/journal.pone.0167292 27902787 PMC5130262

[plb70130-bib-0070] Nozzolillo C. , Amiguet V.T. , Bily A.C. , Harris C.S. , Saleem A. , Andersen Ø.M. , Jordheim M. (2010) Novel aspects of the flowers and floral pigmentation of two cleome species (Cleomaceae), *C. hassleriana* and *C. serrulata* . Biochemical Systematics and Ecology, 38, 361–369. 10.1016/j.bse.2010.03.005

[plb70130-bib-0071] Nyalala S. , Grout B. (2007) African spider flower (*Cleome gynandra* L./*Gynandropsis gynandra* (L.) Briq.) as a red spider mite (*Tetranychus urticae* Koch) repellent in cut‐flower rose (*Rosa hybrida* L.) cultivation. Scientia Horticulturae, 114, 194–198. 10.1016/j.scienta.2007.06.010

[plb70130-bib-0072] Okonwu K. , Ekeke C. , Mensah S. (2017) Micromorphological and phytochemical studies on *Cleome rutidosperma* Linn. Journal of Advances in Biology & Biotechnology, 11, 1–8. 10.9734/JABB/2017/31028

[plb70130-bib-0073] Olaoluwa E.A. , Azeez S.O. (2022) Chromosomal and reproductive barriers among three species of *cleome* L. from Ile‐Ife, Nigeria. Nordic Journal of Botany, 2022, e03534. 10.1111/njb.03534

[plb70130-bib-0074] Oluoch M.O. , Pichop G.N. , Silué D. , Abukutsa‐Onyango M.O. , Diouf M. , Shackleton C.M. (2009) Production and harvesting systems for African indigenous vegetables. In: Shackleton C.M. , Pasquini M. , Drescher A. (Eds), African indigenous vegetables in urban agriculture. Earthscan, London, pp 145–175.

[plb70130-bib-0075] Omondi E.O. , Engels C. , Nambafu G. , Schreiner M. , Neugart S. , Abukutsa‐Onyango M. , Winkelmann T. (2017) Nutritional compound analysis and morphological characterization of spider plant (*Cleome gynandra*) ‐ an African indigenous leafy vegetable. Foodservice Research International, 100, 284–295. 10.1016/j.foodres.2017.06.050 28873690

[plb70130-bib-1011] Onyango C. .M. , Kunyanga C. .N. , Ontita E. .G. , Narla R. .D. , Kimenju J. .W. (2013) Production, utilization and indigenous knowledge of spider plant in Kenya. African Crop Science Proceedings., 11, 925–930.

[plb70130-bib-0076] Osborne C.P. , Sack L. (2012) Evolution of C_4_ plants: a new hypothesis for an interaction of CO_2_ and water relations mediated by plant hydraulics. Philosophical Transactions of the Royal Society, B: Biological Sciences, 367, 583–600. 10.1098/rstb.2011.0261 PMC3248710 22232769

[plb70130-bib-0139] Parma D.F. (2021) Anatomical, Morphophysiological and Molecular Characterization of Brazilian Species of Cleomaceae. Doctoral thesis (Ph.D. in Botany) – Federal University of Viçosa, Viçosa, 204 pp. https://locus.ufv.br/items/a2b4f826-b531-4c51-afb7-596876649683

[plb70130-bib-0077] Parma D.F. , Souza K.F. , Vaz M.G.M.V. , Martins S.B. , Araújo W.L. , Zsögön A. , Weber A.P.M. , Schranz M.E. , Nunes‐Nesi A. (2023) Exploring the diversity of sexual systems and pollination in Brazilian Cleomaceae species. Flora, 300, 152245. 10.1016/j.flora.2023.152245

[plb70130-bib-0078] Parma D.F. , Vaz M.G.M.V. , Falquetto P. , Silva J.C. , Clarindo W.R. , Westhoff P. , Van Velzen R. , Schlüter U. , Araújo W.L. , Schranz M.E. , Weber A.P.M. , Nunes‐Nesi A. (2022) New insights into the evolution of C_4_ photosynthesis offered by the Tarenaya cluster of Cleomaceae. Frontiers in Plant Science, 12, 756505. 10.3389/fpls.2021.756505 35116048 PMC8803641

[plb70130-bib-0079] Patchell M.J. , Bolton M.C. , Mankowski P. , Hall J.C. (2011) Comparative floral development in Cleomaceae reveals two distinct pathways leading to Monosymmetry. International Journal of Plant Sciences, 172, 352–365. 10.1086/658158

[plb70130-bib-0080] Patchell M.J. , Roalson E.H. , Hall J.C. (2014) Resolved phylogeny of Cleomaceae based on all three genomes. Taxon, 63, 315–328. 10.12705/632.17

[plb70130-bib-0081] Pax F. , Hoffm K. (1936) Capparidaceae, Die Natürlichen Pflanzenfamilien, Vol. 17b, 2nd edition. W. Engelmann, Leipzig, pp 146–223.

[plb70130-bib-0082] Prasifka J.R. , Mallinger R.E. , Portlas Z.M. , Hulke B.S. , Fugate K.K. , Paradis T. , Hampton M.E. , Carter C.J. (2018) Using nectar‐related traits to enhance crop‐pollinator interactions. Frontiers in Plant Science, 9, 812. 10.3389/fpls.2018.00812 29967631 PMC6015894

[plb70130-bib-0083] Raghavendra A. , Das V.R. (1978) The occurrence of C_4_‐photosynthesis: a supplementary list of C_4_ plants reported during late 1974‐mid 1977.

[plb70130-bib-1007] Rajendrudu G. , Rama Das V.S. (1982) Biomass production of two species of *Cleome* exhibiting C₃ and C₄ photosynthesis. Biomass, 2, 223–227. 10.1016/0144-4565(82)90032-4

[plb70130-bib-1009] Rassouli E. , Dadras O. .G. , Bina E. , Asgarpanah J. (2014) The essential oil composition of *Cleome brachycarpa* Vahl ex DC. Journal of Essential Oil‐Bearing Plants, 17, 158–163. 10.1080/0972060X.2014.884784

[plb70130-bib-0084] Rathore N.S. , Rathore N. , Shekhawat N.S. (2013) In vitro flowering and seed production in regenerated shoots of *Cleome viscosa* . Industrial Crops and Products, 50, 232–236. 10.1016/j.indcrop.2013.07.032

[plb70130-bib-0085] Rawsthorne S. , Hylton C.M. , Smith A.M. , Woolhouse H.W. (1988) Distribution of photorespiratory enzymes between bundle‐sheath and mesophyll cells in leaves of the C_3_‐C_4_ intermediate species *Moricandia arvensis* (L.) DC. Planta, 176, 527–532. 10.1007/BF00397660 24220949

[plb70130-bib-0086] Reeves G. , Singh P. , Rossberg T.A. , Sogbohossou E.O.D. , Schranz M.E. , Hibberd J.M. (2018) Natural variation within a species for traits underpinning C_4_ photosynthesis. Plant Physiology, 177, 504–512. 10.1104/pp.18.00168 29678862 PMC6001323

[plb70130-bib-0087] Reyna‐Llorens I. , Hibberd J.M. (2017) Recruitment of pre‐existing networks during the evolution of C_4_ photosynthesis. Philosophical Transactions of the Royal Society, B: Biological Sciences, 372, 20160386. 10.1098/rstb.2016.0386 PMC5566883 28808102

[plb70130-bib-0088] Riaz S. , Abid R. , Ali S.A. , Munir I. , Qaiser M. (2019) Morphology and seed protein profile for a new species of the genus *cleome L*. (Cleomaceae) from Pakistan. Acta Botanica Croatica, 78, 102–106.

[plb70130-bib-0089] Roalson E.H. , Hall J.C. , Riser I.J.P. , Cardinal‐McTeague W.M. , Cochrane T.S. , Sytsma K.J. (2015) A revision of generic boundaries and nomenclature in the north American cleomoid clade (Cleomaceae). Phytotaxa, 205, 129. 10.11646/phytotaxa.205.3.1

[plb70130-bib-0090] Rocha D.I. , Monte Bello C.C. , Sobol S. , Samach A. , Dornelas M.C. (2015) Auxin and physical constraint exerted by the perianth promote androgynophore bending in *Passiflora mucronata* L. (Passifloraceae). Plant Biology, 17, 639–646. 10.1111/plb.12295 25524599

[plb70130-bib-0091] Sage R.F. (2004) The evolution of C_4_ photosynthesis. New Phytologist, 161, 341–370. 10.1111/j.1469-8137.2004.00974.x 33873498

[plb70130-bib-0092] Sage R.F. (2016) A portrait of the C_4_ photosynthetic family on the 50th anniversary of its discovery: species number, evolutionary lineages, and Hall of fame. Journal of Experimental Botany, 67, 4039–4056. 10.1093/jxb/erw156 27053721

[plb70130-bib-0093] Sage R.F. (2021) Russ Monson and the evolution of C_4_ photosynthesis. Oecologia, 197, 823–840. 10.1007/s00442-021-04883-1 33661402

[plb70130-bib-0094] Saifullah K. , Williams A. , Adkins S. (2023) Spider Plant (*Cleome gynandra L*.): an Emerging Weed in the Sweet Corn–Brassica Cropping Systematics. Agronomy, 13, 1430. 10.3390/agronomy13051430

[plb70130-bib-0095] Sánchez‐Acebo L. (2005) A phylogenetic study of the new world cleome (Brassicaceae, Cleomoideae). Annals of the Missouri Botanical Garden, 92, 179–201.

[plb70130-bib-0096] Sankhla N. , Ziegler H. , Vyas O.P. , Stichler W. , Trimborn P. (1975) Eco‐physiological studies on Indian arid zone plants: VA screening of some species for the C_4_‐pathway of photosynthetic CO_2_‐fixation. Oecologia, 21, 123–129. 10.1007/BF00345555 28308243

[plb70130-bib-0097] Saunders T.C. , Larridon I. , Baker W.J. , Barrett R.L. , Forest F. , Françoso E. , Maurin O. , Rokni S. , Roalson E.H. (2024) Tangled webs and spider‐flowers: phylogenomics, biogeography, and seed morphology inform the evolutionary history of Cleomaceae. American Journal of Botany, 111, e16399. 10.1002/ajb2.16399 39206557

[plb70130-bib-0098] Schlüter U. , Weber A.P.M. (2016) The road to C_4_ photosynthesis: evolution of a complex trait via intermediary states. Plant and Cell Physiology, 57, 881–889. 10.1093/pcp/pcw009 26893471

[plb70130-bib-0099] Schlüter U. , Weber A.P.M. (2020) Regulation and evolution of C_4_ photosynthesis. Annual Review of Plant Biology, 71, 183–215. 10.1146/annurev-arplant-042916-040915 32131603

[plb70130-bib-0100] Schranz M.E. , Mitchell‐Olds T. (2006) Independent ancient polyploidy events in the sister families Brassicaceae and Cleomaceae. The Plant Cell., 18, 1152–1165. 10.1105/tpc.106.041111 16617098 PMC1456871

[plb70130-bib-0101] Schreier T.B. , Müller K.H. , Eicke S. , Faulkner C. , Zeeman S.C. , Hibberd J.M. (2024) Plasmodesmal connectivity in C_4_ *Gynandropsis gynandra* is induced by light and dependent on photosynthesis. New Phytologist, 241, 298–313. 10.1111/nph.19343 37882365 PMC10952754

[plb70130-bib-0102] Schuler M.L. , Mantegazza O. , Weber A.P.M. (2016) Engineering C_4_ photosynthesis into C_3_ chassis in the synthetic biology age. The Plant Journal, 87, 51–65. 10.1111/tpj.13155 26945781

[plb70130-bib-0103] Scorza L.C. , Dornelas M.C. (2014) Rapid touch‐stimulated movement in the androgynophore of *Passiflora* flowers (subgen. *Decaloba*; sect. *Xerogona*): an adaptation to enhance cross‐pollination? Plant Signaling & Behavio, 9, e27932. 10.4161/psb.27932 PMC4091215 24487079

[plb70130-bib-0104] Shahin S. , Kurup S. , Cheruth A.‐J. , Salem M. (2018) Chemical composition of *cleome amblyocarpa* Barr. & Murb. Essential oils under different irrigation levels in Sandy soils with antioxidant activity. Journal of Essential Oil Bearing Plants, 21(5), 1235–1256. 10.1080/0972060X.2018.1512422

[plb70130-bib-0105] Shi X. , Bloom A. (2021) Photorespiration: the futile cycle? Plants, 10, 908. 10.3390/plants10050908 34062784 PMC8147352

[plb70130-bib-0106] Silva A.P.S.D. , Nascimento Da Silva L.C. , Martins Da Fonseca C.S. , De Araújo J.M. , Correia M.T.D.S. , Cavalcanti M.D.S. , Lima V.L.D.M. (2016) Antimicrobial activity and phytochemical analysis of organic extracts from *Cleome spinosa* Jaqc. Frontiers in Microbiology, 7, e00963. 10.3389/fmicb.2016.00963 PMC4924519 27446005

[plb70130-bib-1008] Silva‐Alvim F. .A. .L. , Chaves Alvim J. , Harvey A. .R. , Blatt M. .R. (2024) Speedy stomata of a C₄ plant correlate with enhanced K⁺ channel gating. Plant, Cell & Environment, 47(3), 817–831. 10.1111/pce.14775 PMC10953386 38013592

[plb70130-bib-0107] Singh H. , Mishra A. , Mishra A.K. (2018) The chemistry and pharmacology of cleome genus: a review. Biomedicine & Pharmacotherapy, 101, 37–48. 10.1016/j.biopha.2018.02.053 29477056

[plb70130-bib-0108] Singh P. , Stevenson S.R. , Dickinson P.J. , Reyna‐Llorens I. , Tripathi A. , Reeves G. , Schreier T.B. , Hibberd J.M. (2023) C_4_ gene induction during de‐etiolation evolved through changes in cis to allow integration with ancestral C_3_ gene regulatory networks. Science Advances, 9, eade9756. 10.1126/sciadv.ade9756 36989352 PMC10058240

[plb70130-bib-0132] Soares Neto R.L. , Thomas W.W. , Barbosa M.R.V. , Roalson E.H. (2018) New combinations and taxonomic notes for *Tarenaya* (Cleomaceae). Acta Botânica Brasílica, 32, 389–397.

[plb70130-bib-0110] Soares Neto R.L. , Thomas W.W. , De Vasconcellos Barbosa M.R. , Roalson E.H. (2020) Diversification of New World Cleomaceae with emphasis on *Tarenaya* and the description of *Iltisiella*, a new genus. Taxon, 69, 321–336. 10.1002/tax.12231

[plb70130-bib-0111] Sommer M. , Bräutigam A. , Weber A.P. (2012) The dicotyledonous NAD malic enzyme C_4_ plant *Cleome gynandra* displays age‐dependent plasticity of C4 decarboxylation biochemistry. Plant Biology, 14, 621–629. 10.1111/j.1438-8677.2011.00539.x 22289126

[plb70130-bib-0112] Stevens P.F. , Davis H.M. (2005) The angiosperm phylogeny website ‐ a tool for reference and teaching in a time of change. Proceedings of the American Society for Information Science and Technology, 42,. 10.1002/meet.14504201249

[plb70130-bib-0113] Subramanian D. , Susheela G. (1988) Cytotaxonomical studies of south Indian Capparidaceae. Cytologia, 53, 679–684. 10.1508/cytologia.53.679

[plb70130-bib-0114] Swift J. , Luginbuehl L.H. , Hua L. , Schreier T.B. , Donald R.M. , Stanley S. , Wang N. , Lee T.A. , Nery J.R. , Ecker J.R. , Hibberd J.M. (2024) Exaptation of ancestral cell‐identity networks enables C_4_ photosynthesis. Nature, 636, 143–150. 10.1038/s41586-024-08204-3 39567684 PMC11618092

[plb70130-bib-0115] Thulin M. , Roalson E.H. (2017) Resurrection of the genus *Rorida* (Cleomaceae), a distinctive Old World segregate of *cleome* . Systematic Botany, 42, 569–577. 10.1600/036364417X695989

[plb70130-bib-0116] Triesch S. , Denton A.K. , Bouvier J.W. , Buchmann J.P. , Reichel‐Deland V. , Guerreiro R.N.F.M. , Busch N. , Schlüter U. , Stich B. , Kelly S. , Weber A.P.M. (2024) Transposable elements contribute to the establishment of the glycine shuttle in *Brassicaceae* species. Plant Biology, 26, 270–281. 10.1111/plb.13601 38168881

[plb70130-bib-0117] Triesch S. , Reichel‐Deland V. , Valderrama Martín J.M. , Melzer M. , Schlüter U. , Weber A.P.M. (2025) Single‐nuclei sequencing of *Moricandia arvensis* reveals bundle sheath cell function in the photorespiratory shuttle of C_3_‐C_4_ intermediate Brassicaceae. Journal of Experimental Botany, eraf245. 10.1093/jxb/eraf245 PMC12587421 40590299

[plb70130-bib-0118] Tsai Y.‐T. , Chen P.‐Y. , To K‐Y (2012) Plant regeneration and stable transformation in the floricultural plant *Cleome spinosa*, a C_3_ plant closely related to the C_4_ plant *C. Gynandra* . Plant Cell Reports, 31, 1189–1198. 10.1007/s00299-012-1240-1 22358374

[plb70130-bib-0119] Uusiku N.P. , Oelofse A. , Duodu K.G. , Bester M.J. , Faber M. (2010) Nutritional value of leafy vegetables of sub‐Saharan Africa and their potential contribution to human health: a review. Journal of Food Composition and Analysis, 23, 499–509. 10.1016/j.jfca.2010.05.002

[plb70130-bib-0120] Van Den Bergh E. , Külahoglu C. , Bräutigam A. , Hibberd J.M. , Weber A.P.M. , Zhu X.‐G. , Eric Schranz M. (2014) Gene and genome duplications and the origin of C_4_ photosynthesis: birth of a trait in the Cleomaceae. Current Plant Biology, 1, 2–9. 10.1016/j.cpb.2014.08.001

[plb70130-bib-0121] Voznesenskaya E.V. , Koteyeva N.K. , Chuong S.D.X. , Ivanova A.N. , Barroca J. , Craven L.A. , Edwards G.E. (2007) Physiological, anatomical and biochemical characterisation of photosynthetic types in genus cleome (Cleomaceae). Functional Plant Biology, 34, 247–267. 10.1071/FP06287 32689352

[plb70130-bib-0122] Williams B.P. , Burgess S.J. , Reyna‐Llorens I. , Knerova J. , Aubry S. , Stanley S. , Hibberd J.M. (2016) An untranslated *cis* ‐element regulates the accumulation of multiple C_4_ enzymes in *Gynandropsis gynandra* mesophyll cells. The Plant Cell, 28, 454–465. 10.1105/tpc.15.00570 26772995 PMC4790868

[plb70130-bib-0123] Wu T. , Solberg S.O. , Yndgaard F. , Chou Y.‐Y. (2018) Morphological patterns in a world collection of *Cleome gynandra* . Genetic Resources and Crop Evolution, 65, 271–283. 10.1007/s10722-017-0529-x

[plb70130-bib-0124] Zenchyzen B. , Acorn J.H. , Merkosky K. , Hall J.C. (2024) Shining a light on UV‐fluorescent floral nectar after 50 years. Science Reports, 14, 11992. 10.1038/s41598-024-62626-7 PMC11128001 38796543

[plb70130-bib-0125] Zhao Y.‐Y. , Lyu M.A. , Miao F. , Chen G. , Zhu X.‐G. (2022) The evolution of stomatal traits along the trajectory toward C_4_ photosynthesis. Plant Physiology, 190, 441–458. 10.1093/plphys/kiac252 35652758 PMC9434244

[plb70130-bib-0126] Zhou H. , Helliker B.R. , Huber M. , Dicks A. , Akçay E. (2018) C_4_ photosynthesis and climate through the lens of optimality. Proceedings of the National Academy of Sciences of the United States of America, 115, 12057–12062. 10.1073/pnas.1718988115 30401739 PMC6255158

[plb70130-bib-0127] Zohoungbogbo H.P.F. , Houdegbe C.A. , Sogbohossou D.E.O. , Tossou M.G. , Maundu P. , Schranz E.M. , Van Deynze A. , Zoundjihekpon J. , Achigan‐Dako E.G. (2018) Andromonoecy in *Gynandropsis gynandra* (L.) Briq. (Cleomaceae) and effects on fruit and seed production. Genetic Resources and Crop Evolution, 65, 2231–9. 10.1007/s10722-018-0687-5

